# Modified Fourier–Galerkin Solution for Aerospace Skin-Stiffener Panels Subjected to Interface Force and Mixed Boundary Conditions

**DOI:** 10.3390/ma12172794

**Published:** 2019-08-30

**Authors:** Renluan Hou, Qing Wang, Jiangxiong Li, Yinglin Ke

**Affiliations:** 1 State Key Laboratory of Fluid Power and Mechatronic System, College of Mechanical Engineering, Zhejiang University, Hangzhou 310027, China; 2 Key Laboratory of Advanced Manufacturing Technology of Zhejiang Province, College of Mechanical Engineering, Zhejiang University, Hangzhou 310027, China

**Keywords:** aircraft stiffened panel deformation, Fourier–Galerkin method, Kirchhoff–Love shell, Euler–Bernoulli beam, mechanical joining, mixed boundary conditions

## Abstract

Aeronautical stiffened panels composed of thin shells and beams are prone to deformation or buckling due to the combined loading, functional boundary conditions and interface forces between joined parts in the assembly processes. In this paper, a mechanical prediction model of the multi-component panel is presented to investigate the deformation propagation, which has a significant effect on the fatigue life of built-up structures. Governing equations of Kirchhoff–Love shell are established, of which displacement expressions are transformed into Fourier series expansions of several introduced potential functions by applying the Galerkin approach. This paper presents an intermediate quantity, concentrated force at the joining interface, to describe mechanical interactions between the coupled components. Based on the Euler–Bernoulli beam theory, unknown intermediate quantity is calculated by solving a 3D stringer deformation equation with static boundary conditions specified on joining points. Compared with the finite element simulation and integrated model, the proposed method can substantially reduce grid number without jeopardizing the prediction accuracy. Practical experiment of the aircraft panel assembly is also performed to obtain the measured data. Maximum deviation between the experimental and predicted clearance values is 0.193 mm, which is enough to meet the requirement for predicting dimensional variations of the aircraft panel assembly.

## 1. Introduction

Stiffened panel structure composed of slender beams and a thin-walled shell is widely applied in the fields of marine and aeronautical engineering since it is lightweight and has high load-bearing capacity. Integrated stiffened panel model, regarded as a single piece structure, is generally exploited for the structural idealization of the built-up construction [1]. An aircraft fuselage panel as the built-up stiffened structure is usually assembled by riveting a skin and the stringers together. Before the riveting operation, the actual position of components (skin and stringer) may differ from the designed dimensions due to deformation resulting from the forces at joining interfaces and clamped boundary conditions with non-zero constraints. Thus, the integrated panel analysis cannot achieve an accurate evaluation of the actual profile. Moreover, numerical computation costs of a large-scale aircraft panel with real boundary conditions are also prohibitive. Therefore, a more efficient substructure model of large built-up panels, taking account of the initial variations, coupled interface force and mixed boundary conditions, should be established to achieve computational cost saving and accurate deformation prediction.

Approaches to model the multi-component structures have received attention from aeronautical engineers and mechanical experts, who focus on continuous mechanical descriptions and discrete element modeling of the beam, shell, or their combinations. The integrated one-piece models are mostly utilized in mechanical research to simplify the actual built-up structures. The Euler–Bernoulli beam (EBB) [2], Timoshenko beam [3,4], and nonlinear beam formulation [5] are usually introduced into the deformation analysis of the structures with slender and flexible components. Displacement formulae of these deformation prediction model are expressed or fitted by the Taylor-type expansion [6], Hermite interpolating polynomials [7] and Chebyshev polynomials [8]. The Kirchhoff shell [9], Reissner–Mindlin shell [10], and higher-order approximation shell theory [11] are employed for exploring the intrinsic mechanisms of thin panels. Although the deformation model generated from the single beam theory or shell theory could reduce the computational complexity, it cannot achieve the coupling analysis of structural interaction between multiple adjacent components.

To deal with the static analysis of complex thin-walled structures, Zappino and Carrera [12] built the variable kinematic models using Carrera Unified Formulation, which allows one-, two-, and three-dimensional refined structures to be connected to each other easily. Guida et al. [13] performed a finite element (FE) simulation of the lower lobe fuselage section and also conducted a drop experiment on small components for analyzing the energy absorbing ability of multi-component structures during a crash landing. The grid design for finite element method (FEM) has considerable effects on the time consumption and accuracy of results. Silva et al. [14] proposed a wave finite element method to express the dynamic stiffness matrix of the periodic structures with substructuring techniques that could yield large computational savings. Pacheco et al. [15] established aerodynamic and structural models of reinforced panels based on the principle of virtual work, which is discretized through the FE method. They found that modeling stiffeners as immovable boundaries would overestimate flutter onset conditions and underestimate post-flutter amplitudes. Thus, considering the flexibility of stiffeners is essential to the nonlinear analysis of complex aircraft configurations.

In addition, the solution of boundary value problems for the aforementioned complex mechanical system is another important challenge of multi-component structure analysis. Wesley et al. [16] solved the one- and two-dimensional structural mechanics problems by employing the Lagrange multiplier method and double exponential transformation. Tamijani and Kapania [1] utilized the element-free Galerkin approach to solving the buckling and static problems of simply supported plates with different stiffener configurations. Sapountzakis and Mokos [17] carried out a general solution to the nonlinearly coupled deflection equation of plates stiffened by doubly symmetric cross-sectional beam based on the analog equation method and boundary element method. Ahmad and Kapania [18] presented a vibration solution of integrally stiffened plates with plate-strip stiffeners with the Rayleigh–Ritz approach, which can also be applied to fully clamped, free, and cantilever supported stiffened plates. However, deformation investigations of multi-component structures, such as the previously mentioned ones, usually take no account of the initial variations. For the complex multi-component aircraft, a mechanical model with no consideration of the actual variation of boundary conditions may directly affect the aerodynamic and fatigue performance analysis of the structure.

To meet the computational saving and accuracy requirements for the numerical calculations of a large-scale aircraft stiffened panel, an efficient semi-analytical solution for mechanical substructure model of the joined beam-shell structure is firstly proposed in this paper. Based on the Kirchhoff–Love shell theory, governing equations with displacement and rotation boundary constraints are established to calculate the skin deformation resulting from the initial positioning variations and the coupling interaction between the joined parts. The solution of the shell equations is derived from the Fourier–Galerkin approach. Simultaneously, the displacement formulas of modified Euler–Bernoulli beam are developed to predict the 3D deformation of stringers caused by the positioning errors and clamping operation. For describing the interactions of coupled beam-shell structures, concentrated forces expressed as Dirac delta functions and the static boundary conditions at joining interfaces are introduced into the deformation equations. Moreover, the displacements solutions of the deformed stiffened panel are derived by adopting the Wronskian matrix and potential functions with Fourier series expansions. Finally, the accuracy and efficiency of the proposed semi-analytical method are verified by making a comparison with FE analysis results and the measured data in the practical experiment of the aircraft panel assembly.

## 2. Governing Equations of Stiffened Panel and Its Solution Procedure

**Figure 1:**
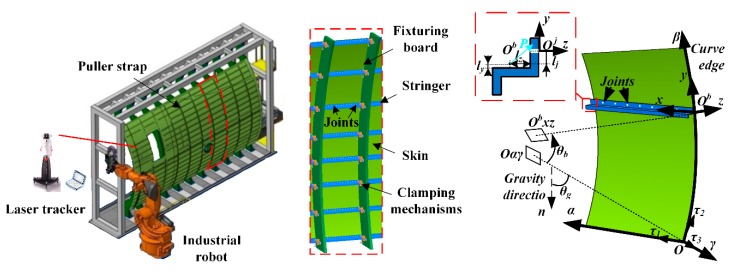
Assembled aircraft stiffened panel structure.

As a typical subassembly part of aircraft, the fuselage stiffened panel is the combination of stringers and skin, as shown in Figure 1. Skin and stiffeners are first positioned and fastened to the specified location on the fixturing boards by adjusting the clamping mechanisms. Position error and the clamping variations on the contact edges could cause the initial deformations of panel components, which are the boundary value problems for governing equations of shell and beam with functional boundary conditions. Subsequently, the joining operation also induces the deflection due to the mechanical interactions between the coupled structures.

### 2.1. Shell Subjected to Concentrated Force at Joining Interface and Functional Boundary Conditions

Kirchhoff–Love shell theory is utilized to establish the deformation model of single curvature skin, regarded as a thin cylindrical shell. The curvilinear coordinate system of the undeformed shell is denoted as *Oαβγ*. Curve edges of the panel are located on fixturing boards, which induce the position variations on boundary conditions, as shown in Figure 1.

The equilibrium equations of cylindrical shell [11] along axes of local coordinate system *Oτ_1_τ_2_τ_3_* are established by

(1) $$\{\begin{array}{c} \frac{\partial N_{1}}{\partial \alpha }+\frac{\partial N_{21}}{\partial \beta }+q_{1}^{}=0\\ \frac{\partial N_{12}}{\partial \alpha }+\frac{\partial N_{2}}{\partial \beta }+\frac{1}{R}\left(\frac{\partial M_{12}}{\partial \alpha }+\frac{\partial M_{2}}{\partial \beta }\right)+q_{2}^{}=0\\ -\frac{N_{2}}{R}+\frac{\partial }{\partial \alpha }\left(\frac{\partial M_{1}}{\partial \alpha }+\frac{\partial M_{21}}{\partial \beta }\right)+\frac{\partial }{\partial \beta }\left(\frac{\partial M_{12}}{\partial \alpha }+\frac{\partial M_{2}}{\partial \beta }\right)+q_{3}^{}=0 \end{array},$$

where $N_{12}=N_{21}$, $M_{12}=M_{21}$. Moreover, the third terms of second equation in Equation (1) are small enough quantities to be eliminated for thin shell [19]. The stress components are expressed in terms of the middle surface stain components, as follows:

(2) $${\left[\begin{array}{ccc} \sigma_{1} & \sigma_{2} & \sigma_{12} \end{array}\right]}^{T}=\left[\begin{array}{cc} Q_{3\times 3}^{I} & Q_{3\times 3}^{I} \end{array}\right]\left[\begin{array}{cc} E_{3\times 3} & 0\\ 0 & \gamma E_{3\times 3} \end{array}\right]{\left[\begin{array}{cccccc} \varepsilon_{1}^{0} & \varepsilon_{2}^{0} & \varepsilon_{12}^{0} & \chi_{1} & \chi_{2} & \chi_{12} \end{array}\right]}^{T},$$

where $\left[E_{3\times 3}\right]$ is a unit matrix, elements of matrix [**Q^I^**] are $Q_{11}=Q_{22}=\frac{E}{1-\mu^{2}}$, $Q_{12}=Q_{21}=\frac{E\mu }{1-\mu^{2}}$, $Q_{33}=\frac{E}{2(1+\mu )}$, other elements of [**Q^I^**] are zeroes, strains are $\varepsilon_{1}^{0}=\frac{\partial u}{\partial \alpha }$, $\varepsilon_{2}^{0}=\frac{\partial v}{\partial \beta }+\frac{w}{R}$ and $\varepsilon_{12}^{0}=\frac{\partial v}{\partial \alpha }+\frac{\partial u}{\partial \beta }$, changes in curvature and twist are $\chi_{1}=-\frac{\partial^{2}w}{\partial \alpha^{2}}$, $\chi_{2}=-\frac{\partial^{2}w}{\partial \beta^{2}}$ and $\chi_{12}=-2\frac{\partial^{2}w}{\partial \alpha \partial \beta }$. The stress resultants and stress couples of a thin cylindrical shell are given by:

(3) $${\left[\begin{array}{cccccc} N_{1} & N_{2} & N_{12} & M_{1} & M_{2} & M_{12} \end{array}\right]}^{T}=\int_{-h/2}^{h/2}\left[\begin{array}{c} E_{3\times 3}\\ \gamma E_{3\times 3} \end{array}\right]\left[\begin{array}{c} \sigma_{1}\\ \sigma_{2}\\ \sigma_{12} \end{array}\right]d\gamma =\left[Q^{*}\right]{\left[\begin{array}{cccccc} \varepsilon_{1}^{0} & \varepsilon_{2}^{0} & \varepsilon_{12}^{0} & \chi_{1} & \chi_{2} & \chi_{12} \end{array}\right]}^{T}$$

where $\left[Q^{*}\right]=\int_{-h/2}^{h/2}\left[\begin{array}{c} E_{3\times 3}\\ \gamma E_{3\times 3} \end{array}\right]\left[\begin{array}{cc} Q_{3\times 3}^{I} & Q_{3\times 3}^{I} \end{array}\right]\left[\begin{array}{cc} E_{3\times 3} & 0\\ 0 & \gamma E_{3\times 3} \end{array}\right]d\gamma =\left[\begin{array}{cc} hQ_{}^{I} & 0\\ 0 & (h^{3}/12)Q_{}^{I} \end{array}\right]$.

Substituting Equation (3) into Equation (1), governing equations of the deformation of a thin cylindrical shell can be derived as

(4) $$\left[D\right]\left[\begin{array}{c} u\\ v\\ w \end{array}\right]+\frac{1-\mu^{2}}{Eh}\left[\begin{array}{c} q_{1}^{}\\ q_{2}^{}\\ -q_{3}^{} \end{array}\right]=\left[\begin{array}{c} 0\\ 0\\ 0 \end{array}\right].$$

Differential operator matrix of the above equation is denoted by:

(5) $$D=\left[\begin{array}{ccc} \frac{\partial^{2}}{\partial \alpha^{2}}+\frac{1-\mu }{2}\frac{\partial^{2}}{\partial \beta^{2}} & \frac{1+\mu }{2}\frac{\partial^{2}}{\partial \alpha \partial \beta } & \frac{\mu }{R}\frac{\partial }{\partial \alpha }\\ \frac{1+\mu }{2}\frac{\partial^{2}}{\partial \alpha \partial \beta } & \frac{\partial^{2}}{\partial \beta^{2}}+\frac{1-\mu }{2}\frac{\partial^{2}}{\partial \alpha^{2}} & \frac{1}{R}\frac{\partial }{\partial \beta }\\ \frac{\mu }{R}\frac{\partial }{\partial \alpha } & \frac{1}{R}\frac{\partial }{\partial \beta } & \frac{1}{R^{2}}+\frac{h^{2}}{12}\left(\frac{\partial^{4}}{\partial \alpha^{4}}+2\frac{\partial^{4}}{\partial \alpha^{2}\partial \beta^{2}}+\frac{\partial^{4}}{\partial \beta^{4}}\right) \end{array}\right].$$

Since the symmetric matrix [D] is a linear differential operator matrix, Equation (5) can be transformed into the combination of several differential equations of potential functions. The introduced potential functions $\Phi_{i}$ (i=1,2,3) have the following relations with displacements:

(6) $$\left[\begin{array}{c} u\\ v\\ w \end{array}\right]=\left[\begin{array}{ccc} \left|D_{11}\right| & \left|D_{12}\right| & \left|D_{13}\right|\\ \left|D_{21}\right| & \left|D_{22}\right| & \left|D_{23}\right|\\ \left|D_{31}\right| & \left|D_{32}\right| & \left|D_{33}\right| \end{array}\right]\left[\begin{array}{c} \Phi_{1}\\ \Phi_{2}\\ \Phi_{3} \end{array}\right],$$

where $\left|D_{ij}\right|$ (i,j=1,2,3) is the cofactor of determinant |D|. Substituting Equation (6) into Equation (4) yields:

(7) $$\left[\begin{array}{ccc} \left|D\right| & 0 & 0\\ 0 & \left|D\right| & 0\\ 0 & 0 & \left|D\right| \end{array}\right]\left[\begin{array}{c} \Phi_{1}\\ \Phi_{2}\\ \Phi_{3} \end{array}\right]+\left[\begin{array}{c} q_{1}^{}\\ q_{2}^{}\\ -q_{3}^{} \end{array}\right]=\left[\begin{array}{c} 0\\ 0\\ 0 \end{array}\right].$$

The Galerkin approach is employed to derive solutions of the governing equations. According to the expressions satisfying the simply supported boundary conditions prescribed on the two straight edges of shell in [20], displacement and rotation components are assumed as:

(8) $${\left[\begin{array}{ccc} u(\alpha ,\beta ) & v(\alpha ,\beta ) & w(\alpha ,\beta ) \end{array}\right]}^{T}=\sum_{m=0}^{\infty }\left[T_{m}(\beta )\right]{\left[\begin{array}{ccc} U(\alpha ) & V(\alpha ) & W(\alpha ) \end{array}\right]}^{T},$$

where $\left[T_{m}(\beta )\right]=\left[\begin{array}{ccc} \sin\bar{m}\beta & 0 & 0\\ 0 & \cos\bar{m}\beta & 0\\ 0 & 0 & \sin\bar{m}\beta \end{array}\right]$, $\bar{m}=m\pi /b$. The displacements and stress acting on the straight edges of shell, $\beta_{1}=0$ and $\beta_{2}=b$, have the following relationships:

(9) $$u{|}_{\beta =\beta_{i}}=w{|}_{\beta =\beta_{i}}=0,\;N_{2}{|}_{\beta =\beta_{i}}=\frac{E^{k}h}{1-\mu^{2}}{\left(\frac{\partial v}{\partial \beta }+\frac{w}{R}+\mu \frac{\partial u}{\partial \alpha }\right)|}_{\beta =\beta_{i}}=0,\;M_{2}{|}_{\beta =\beta_{i}}=-\frac{E^{k}h^{3}}{12\left[1-\mu^{2}\right]}{\left(\frac{\partial^{2}w}{\partial \beta^{2}}+\mu \frac{\partial^{2}w}{\partial \alpha^{2}}\right)|}_{\beta =\beta_{i}}=0,\;(i=1,2),$$

which are also the boundary conditions on straight edges of panel.

Therefore, the variables in potential functions and the loads are decoupled by expanding the functions into the form of Fourier series as Equation (8):

(10) $${\left[\begin{array}{ccc} \Phi_{1}\left(\alpha ,\beta \right) & \Phi_{2}\left(\alpha ,\beta \right) & \Phi_{3}\left(\alpha ,\beta \right) \end{array}\right]}^{T}=\sum_{m=0}^{\infty }\left[T_{m}(\beta )\right]{\left[\begin{array}{ccc} \Phi_{1}^{*}\left(\alpha \right) & \Phi_{2}^{*}\left(\alpha \right) & \Phi_{3}^{*}\left(\alpha \right) \end{array}\right]}^{T},$$

and

(11) $${\left[\begin{array}{ccc} q_{1}\left(\alpha ,\beta \right) & q_{2}\left(\alpha ,\beta \right) & q_{3}\left(\alpha ,\beta \right) \end{array}\right]}^{T}=\sum_{m=0}^{\infty }\left[T_{m}(\beta )\right]{\left[\begin{array}{ccc} q_{1m}(\alpha ) & q_{2m}(\alpha ) & q_{3m}(\alpha ) \end{array}\right]}^{T},$$

where

(12) $${\left[\begin{array}{ccc} q_{1m} & q_{2m} & q_{3m} \end{array}\right]}^{T}=\frac{c_{m}}{b}\int_{0}^{b}\left[T_{m}(\beta )\right]{\left[\begin{array}{ccc} q_{{}_{1}}^{}\left(\alpha ,\beta \right) & q_{{}_{2}}^{}\left(\alpha ,\beta \right) & q_{{}_{3}}^{}\left(\alpha ,\beta \right) \end{array}\right]}^{T}d\beta ,\;\;c_{m}=\{\begin{array}{c} 1\\ 2 \end{array}\begin{array}{cc} & \begin{array}{c} m=0\\ m\neq 0 \end{array} \end{array}.$$

Substituting Equations (8) and (11) into Equation (4), the displacements and loads with m=0 can be rewritten as:

(13) $$u=0,\;w=0,\;q_{1}^{}=0,\;q_{3}^{}=0,\;q_{2}^{}=q_{2m}(\alpha ),\;D_{22}V(\alpha )+q_{2m}(\alpha )=0.$$

where v=V(α)=−1Q33*∬q2m(α)dα2+J01α+J02, J0i(i=1,2) is the coefficient to be determined. When m≠0, substituting the expanded Equations (10)–(12) into Equation (7) and eliminating the trigonometric terms, the following equations can be obtained:

(14) $$G\left[\begin{array}{ccccc} 1 & -4{\bar{m}}^{2} & 6{\bar{m}}^{4}+\frac{12(1-\mu^{2})}{h^{2}R^{2}} & -4{\bar{m}}^{6} & {\bar{m}}^{8} \end{array}\right]{\left[\begin{array}{ccccc} \frac{d^{8}\Phi_{i}^{*}}{d\alpha^{8}} & \frac{d^{6}\Phi_{i}^{*}}{d\alpha^{6}} & \frac{d^{4}\Phi_{i}^{*}}{d\alpha^{4}} & \frac{d^{2}\Phi_{i}^{*}}{d\alpha^{2}} & \Phi_{i}^{*} \end{array}\right]}^{T}+\iota q_{im}=0,\;\;\iota =\{\begin{array}{c} \begin{array}{cc} 1 & \begin{array}{cc} for & i=1,2 \end{array} \end{array}\\ \begin{array}{cc} -1 & \begin{array}{cc} for & i=3, \end{array} \end{array} \end{array}$$

where constant $G=\frac{Eh^{3}}{24\left(1+\mu \right)}$. The solution $\begin{array}{cc} \Phi_{i}^{*} & \left(i=1,\;2,\;3\right) \end{array}$ of Equation (14) must be derived to calculate $\begin{array}{cc} \Phi_{i} & \left(i=1,2,3\right) \end{array}$, which is the functions in the displacement Equation (6), i.e., the solutions of shell deformation equation.

The complete solution $\Phi_{i}^{*}$ is the sum of the homogeneous solution and the particular solution. Firstly, the solutions of the homogeneous equation corresponding to Equation (14) are calculated by solving the characteristic equation:

(15) $$\left[\begin{array}{ccccc} 1 & -4{\bar{m}}^{2} & 6{\bar{m}}^{4}+\frac{12(1-\mu^{2})}{h^{2}R^{2}} & -4{\bar{m}}^{6} & {\bar{m}}^{8} \end{array}\right]{\left[\begin{array}{ccccc} \eta^{8} & \eta^{6} & \eta^{4} & \eta^{2} & 1 \end{array}\right]}^{T}=0.$$

All roots of Equation (15) are obtained by:

(16) $${\left[\begin{array}{cccccccc} \eta_{1} & \eta_{2} & \eta_{3} & \eta_{4} & \eta_{5} & \eta_{6} & \eta_{7} & \eta_{8} \end{array}\right]}^{T}=\left[\begin{array}{cc} Y_{4\times 2} & 0\\ 0 & Y_{4\times 2} \end{array}\right]\left[\begin{array}{cccc} \xi_{1} & \xi_{2} & \xi_{3} & \xi_{4} \end{array}\right],\;\;Y_{4\times 2}={\left[\begin{array}{cccc} 1 & -1 & 1 & -1\\ i & -i & -i & i \end{array}\right]}^{T}$$

where real and imaginary parts $\xi_{j}$ (j=1,2,3,4) of complex roots $\eta_{j}$ (j=1,2,…8) are listed as Equation (A1) in Appendix A. Solutions of the homogeneous equation corresponding to Equation (14) can be expressed by:

(17) Φhi*(α)=[eαξ1eαξ3e−αξ3e−αξ1][S][Jm]

where elements of [S] are $S_{11}=S_{47}=\cos(\alpha \xi_{2})$, $S_{12}=-S_{48}=\sin(\alpha \xi_{2})$, $S_{23}=S_{35}=\cos(\alpha \xi_{4})$, $S_{24}=-S_{36}=\sin(\alpha \xi_{4})$ and other elements of [S] are zeroes, [Jm]=[Jm1Jm2Jm3Jm4Jm5Jm6Jm7Jm8]T is a set of real coefficients in displacement expressions to be determined.

Secondly, the particular solutions of Equation (14) are derived by using the Wronskian determinant. The Wronskian determinant of the functions $e^{\eta_{n}\alpha }$ (n=1,2,…8), a fundamental set of solutions to the associated homogeneous equation to Equation (14), is defined by:

(18) $$\left|W\right|=e^{\alpha \sum_{n=1}^{8}\eta_{n}}\prod_{n=1}^{7}\prod_{j=n+1}^{8}\left(\eta_{n}-\eta_{j}\right).$$

The determinant $\left|W_{j}\right|$ is denoted by replacing the *j-*th column of the Wronskian with the column (0,0,…,0,1)^T^.

Particular solutions of Equation (14) can be derived from the following expression:

(19) Φpi*(α)=∑j=18{eηjα∫[−ιGqim]|Wj||W|dα}.

The load functions of concentrated force and uniform load encountered in the practical joining process of aircraft panel assembly are introduced to derive the explicit expressions of Eqaution (19). When the concentrated force *f* is imposed on the joining interface of shell at point $\left(\alpha_{p},\beta_{p}\right)$, the associated distributed load *q* introduced into the deformation equation is expressed as the Dirac delta function $f_{}\delta (\alpha -\alpha_{p})\delta (\beta -\beta_{p})$. Using Equation (12), the following expression can be derived:

(20) $${\left[\begin{array}{ccc} q_{1m}(\alpha ) & q_{2m}(\alpha ) & q_{3m}(\alpha ) \end{array}\right]}^{T}=\frac{c_{m}}{b}\delta (\alpha -\alpha_{p})\int_{0}^{b}\delta (\beta -\beta_{p})\left[T_{m}(\beta_{p})\right]{\left[\begin{array}{ccc} f_{1} & f_{2} & f_{3} \end{array}\right]}^{T}$$

Then, the particular solution of Equation (14) with concentrated force applied on shell when m≠0 is transformed as:

(21) [Φconp1*Φconp2*Φconp3*]T=2Gb∑j=18e(α−αp)ηj∏s=1j−1(ηj−ηs)∏s=j+18(ηj−ηs)H(α−αp)[Tm(βp)][f1f2f3]T.

When the uniform load is imposed on the shell, the particular solution of Equation (14) with m≠0 can be easily obtained by:

(22) [Φunip1*Φunip2*Φunip3*]T=−ιGm¯8[q1mq2mq3m]T

where *q_im_* is a constant or linear function of *α*.

Since the distributed load is a combination of concentrated force and uniform load, the complete solution of Equation (14) with m≠0 should be the superposition of Equations (17), (21) and (22) as Φi*=Φhi*+Φpi*=Φhi*+Φunipi*+Φconpi*. Subsequently, substituting $\Phi_{i}^{*}$ into Equations (10) and (6), the displacements expressions with coefficients Jmi(i=1,2...8) can be obtained as Equation (A2).

Moreover, the presented displacement functions should satisfy the boundary conditions prescribed on shell edges. The initial variations on the skin curve edges are regarded as the non-zero kinematic boundary conditions imposed on edges $\alpha =\alpha_{i}$ (i=1,2), as follows:

(23) $$u{|}_{\alpha =\alpha_{i}}=U_{i}(\beta ),\;v{|}_{\alpha =\alpha_{i}}=V_{i}(\beta ),\;w{|}_{\alpha =\alpha_{i}}=W_{i}(\beta ),\;\varphi_{v}{|}_{\alpha =\alpha_{i}}=\Psi V_{i}(\beta ),$$

where $\varphi_{v}=-\partial w/\partial \alpha$ is the rotation around the axis *β*. Expanding $U_{i}(\beta )$, $V_{i}(\beta )$, $W_{i}(\beta )$ and $\Psi V_{i}(\beta )$ into Fourier series as the form of displacements expressions in Equations (A3) and (A4), a set of linear algebraic equations specific to coefficients are generated. Then, coefficients Jmi(i=1,2...8) can be solved. Deformation of arbitrary point (*α,β,γ*) on the shell is determined by $u^{\gamma }(\alpha ,\beta ,\gamma )=u(\alpha ,\beta )+\varphi_{v}\gamma$, $v^{\gamma }(\alpha ,\beta ,\gamma )=v(\alpha ,\beta )-\varphi_{u}\gamma$ and $w^{\gamma }(\alpha ,\beta ,\gamma )=w(\alpha ,\beta )$. Eventually, the semi-analytical expression of shell deformation with concentrated force, uniform load and arbitrary functional boundary conditions applied are achieved.

### 2.2. Fundamental Equation of Spatial Stiffener with Arbitrary Boundaries

Deformation of the stiffener is analyzed by the modified EBB model. Modified EBB approach introduces the transformation relations of displacements and rotations between arbitrary points on the same cross-section of the stringer entity. Cartesian coordinate system *O^b^xyz* of beam is shown in Figure 1. $\varphi_{x}$, $\varphi_{y}=-dw^{b}/dx$ and $\varphi_{z}=dv^{b}/dx$ are the rotations around three coordinate axes, respectively.

With the assumptions imposed in EBB theory, the stress resultants can be described as [21,22]:

(24) $${\left[\begin{array}{cccc} N_{x} & M_{y} & M_{z} & M_{yz} \end{array}\right]}^{T}=\left[B\right]{\left[\begin{array}{cccc} \varepsilon_{xx} & K_{y} & K_{z} & K_{x} \end{array}\right]}^{T},$$

where elements of matrix **[B]** are $B_{11}=E^{b}A$, $B_{22}=E^{b}I_{yy}$, $B_{23}=B_{32}=E^{b}I_{yz}$, $B_{33}=E^{b}I_{zz}$, $B_{44}=\frac{E^{b}I_{xx}}{2(1+\mu^{b})}$, other elements of **[B]** are zeroes, $\varepsilon_{xx}^{}=du^{b}/dx$ is the axial strain, $K_{x}=d\varphi_{x}/dx$, $K_{y}=-d^{2}w^{b}/dx^{2}$ and $K_{z}=d^{2}v^{b}/dx^{2}$ denote components of the curvature vector of axis *x*.

The governing differential equations of EBB are derived based on the principle of minimum total potential energy, which implies that the total potential energy of the system must be stationary and the stationary value is always a minimum. The total potential energy of the system is defined by:

(25) $$\Gamma (u^{b},v^{b},w^{b})=\frac{1}{2}S_{\Sigma }(u^{b},v^{b},w^{b})-\Theta_{\Sigma }(u^{b},v^{b},w^{b}),$$

where $\Gamma (u^{b},v^{b},w^{b})$ is a functional, $\frac{1}{2}S_{\Sigma }(u^{b},v^{b},w^{b})$ is total strain energy, $\Theta_{\Sigma }(u^{b},v^{b},w^{b})$ is the work done by external forces. To achieve the stationary total potential energy, the variation of Equation (25) should satisfy the following relations:

(26) $$\delta \Gamma =\delta S_{\Sigma }-\delta \Theta_{\Sigma }=0.$$

The strain energy of a beam is defined in terms of six components: one axial force, two bending moments, two shear forces and one torsional moment [23]. Since the shear deformation is neglected in the EBB theory, the total strain energy is expressed by the sum of the strain energy components due to tension or compression, bending and torsion force. The variation of the strain energy is described as:

(27) $$\delta S_{\Sigma }=\int \left(N_{x}\delta \varepsilon_{xx}^{}+M_{y}\delta K_{y}+M_{z}\delta K_{z}+M_{yz}\delta K_{x}\right)dx.$$

The virtual work of external forces is given by:

(28) $$\delta \Theta_{\Sigma }=\int \left(q_{x}\delta u^{b}+q_{rx}\delta \varphi_{x}\right)dx+\int_{x_{1}}^{x_{2}}\left(q_{y}\delta v^{b}+q_{z}\delta w^{b}\right)dx+{\left[q_{y}\delta v^{b}+q_{z}\delta w^{b}+q_{ry}\delta \varphi_{y}+q_{rz}\delta \varphi_{z}\right]}_{x_{1}}^{x_{2}},$$

where the notation ${\left[\ldots \right]}_{x_{1}}^{x_{2}}$ means the minus of the function values with selected independent variable values *x_2_* and *x_1_*. Using the Green’s theorem and method of integration by parts, Equation (26) is rewritten in the following form:

(29) $$\begin{array}{ll} \delta S_{\Sigma }-\delta \Theta_{\Sigma }= & -\int_{x_{1}}^{x_{2}}\left[\frac{dN_{x}}{dx}+q_{x}\right](\delta u^{b})dx+{\left[\left(N_{x}-q_{x})(\delta u^{b}\right)\right]}_{x_{1}}^{x_{2}}-\int_{x_{1}}^{x_{2}}\left[\frac{dM_{yz}}{dx}+q_{rx}\right](\delta \varphi_{x})dx+{\left[\left(M_{yz}-q_{rx})(\delta \varphi_{x}\right)\right]}_{x_{1}}^{x_{2}}\\ & +\int_{x_{1}}^{x_{2}}\left[\frac{d^{2}M_{z}}{dx^{2}}-q_{y}\right](\delta v^{b})dx-\int_{x_{1}}^{x_{2}}\left[\frac{d^{2}M_{y}}{dx^{2}}+q_{z}\right](\delta w^{b})dx+{\left[(M_{z}-q_{rz})\frac{d(\delta v^{b})}{dx}\right]}_{x_{1}}^{x_{2}}-{\left[(M_{y}-q_{ry})\frac{d(\delta w^{b})}{dx}\right]}_{x_{1}}^{x_{2}}\\ & -{\left[(\frac{dM_{z}}{dx}+q_{y})\delta v^{b}\right]}_{x_{1}}^{x_{2}}+{\left[(\frac{dM_{y}}{dx}-q_{z})\delta w^{b}\right]}_{x_{1}}^{x_{2}}\\ & =0. \end{array}$$

Simplifying Equation (29), the governing equations and the static boundary conditions are derived as:

(30) $${\left[\begin{array}{cccc} \frac{dN_{x}}{dx} & \frac{d^{2}M_{y}}{dx^{2}} & \frac{d^{2}M_{z}}{dx^{2}} & \frac{dM_{yz}}{dx} \end{array}\right]}^{T}+{\left[\begin{array}{cccc} q_{x} & q_{z} & -q_{y} & q_{rx} \end{array}\right]}^{T}=0,$$

(31) $$\begin{array}{cccccc} {N_{x}|}_{x=x_{i}}={q_{x}|}_{x=x_{i}}, & {M_{y}|}_{x=x_{i}}={q_{ry}|}_{x=x_{i}}, & {M_{z}|}_{x=x_{i}}={q_{rz}|}_{x=x_{i}}, & {\frac{dM_{y}}{dx}|}_{x=x_{i}}={q_{z}|}_{x=x_{i}}, & {\frac{dM_{z}}{dx}|}_{x=x_{i}}=-{q_{y}|}_{x=x_{i}}, & {M_{yz}|}_{x=x_{i}}={q_{rx}|}_{x=x_{i}}, \end{array}\;(i=1,2).$$

The governing equation of EBB is expressed by substituting stress resultants–strain relation Equation (24) into Equation (30), as follows:

(32) $$\left[B\right]{\left[\begin{array}{cccc} \frac{d^{2}u^{b}}{dx^{2}} & \frac{d^{4}v^{b}}{dx^{4}} & \frac{d^{4}w^{b}}{dx^{4}} & \frac{d^{2}\varphi_{x}}{dx^{2}} \end{array}\right]}^{T}={\left[\begin{array}{cccc} q_{x} & q_{y} & q_{z} & q_{rx} \end{array}\right]}^{T}$$

The position error and clamping variations on the edges of stiffeners are regarded as the following kinematic boundary conditions:

(33) $$\{\begin{array}{c} \begin{array}{ccc} u^{b}{|}_{x=x_{i}}=u_{i}^{b}, & v^{b}{|}_{x=x_{i}}=v_{i}^{b}, & w^{b}{|}_{x=x_{i}}=w_{i}^{b} \end{array}\\ \begin{array}{ccc} \varphi_{x}{|}_{x=x_{i}}=\varphi_{xi}, & \varphi_{y}{|}_{x=x_{i}}=\varphi_{yi}, & \varphi_{z}{|}_{x=x_{i}}=\varphi_{zi} \end{array} \end{array}(i=1,2).$$

For each joining point on the cross section of the beam $x=x_{p}$, eight supplementary boundary conditions should be added into the EBB deformation equation as:

(34) $$\{\begin{array}{cccc} -{\left[M_{y}\right]}_{x_{p}-0}^{x_{p}+0}=0, & {\left[M_{z}\right]}_{x_{p}-0}^{x_{p}+0}=0, & {\left[\frac{dM_{z}}{dx}\right]}_{x_{p}-0}^{x_{p}+0}=f_{y}, & -{\left[\frac{dM_{y}}{dx}\right]}_{x_{p}-0}^{x_{p}+0}=f_{z},\\ v^{b}{|}_{x_{p}+0}=v^{b}{|}_{x_{p}-0}, & w^{b}{|}_{x_{p}+0}=w^{b}{|}_{x_{p}-0}, & \varphi_{y}{|}_{x_{p}+0}=\varphi_{y}{|}_{x_{p}-0}, & \varphi_{z}{|}_{x_{p}+0}=\varphi_{z}{|}_{x_{p}-0}, \end{array}$$

Thus, displacements *u^b^*, *v^b^* and *w^b^*, the piecewise functions with unknown coefficient *C_j_*, could be expressed by the polynomial functions of independent variable *x* since the derived governing equation is a set of the linear partial differential equation.

In particular, the modified EBB method introduces the transformation relations between the displacements of the centroid and arbitrary joining point on the cross-section of the stringer, the following expression [24] is presented by:

(35) $${\left[u^{st},v^{st},w^{st}\right]}^{T}=\left[\Psi \left(\varphi_{x},\varphi_{y},\varphi_{z}\right)-E\right]P+{\left[u^{b},v^{b},w^{b}\right]}^{T},$$

where the coordinate of arbitrary joining point on the cross-section $x=x_{p}$ with respect to Cartesian coordinate system *O^b^xyz* is $P={\left[0,y_{p},z_{p}\right]}^{T}$, rotation matrix $\Psi (\varphi_{x},\varphi_{y},\varphi_{z})=\left[\begin{array}{ccc} \cos\varphi_{z}\cos\varphi_{y} & \cos\varphi_{z}\sin\varphi_{y}\sin\varphi_{x}-\sin\varphi_{z}\cos\varphi_{x} & \cos\varphi_{z}\sin\varphi_{y}\cos\varphi_{x}+\sin\varphi_{z}\sin\varphi_{x}\\ \sin\varphi_{z}\cos\varphi_{y} & \sin\varphi_{z}\sin\varphi_{y}\sin\varphi_{x}+\cos\varphi_{z}\cos\varphi_{x} & \sin\varphi_{z^{O}}\cdot \sin\varphi_{y}\cos\varphi_{x}-\cos\varphi_{z}\sin\varphi_{x}\\ -\sin\varphi_{y} & \cos\varphi_{y}\sin\varphi_{x} & \cos\varphi_{y}\cos\varphi_{x} \end{array}\right]$.

Consequently, the displacements and rotations of arbitrary points on the deformed stringer with positioning and clamping variations and the mechanical joining interaction are derived as Equations (32) and (35).

### 2.3. Calculation Procedure for Stiffened Panel deformation

**Figure 2:**
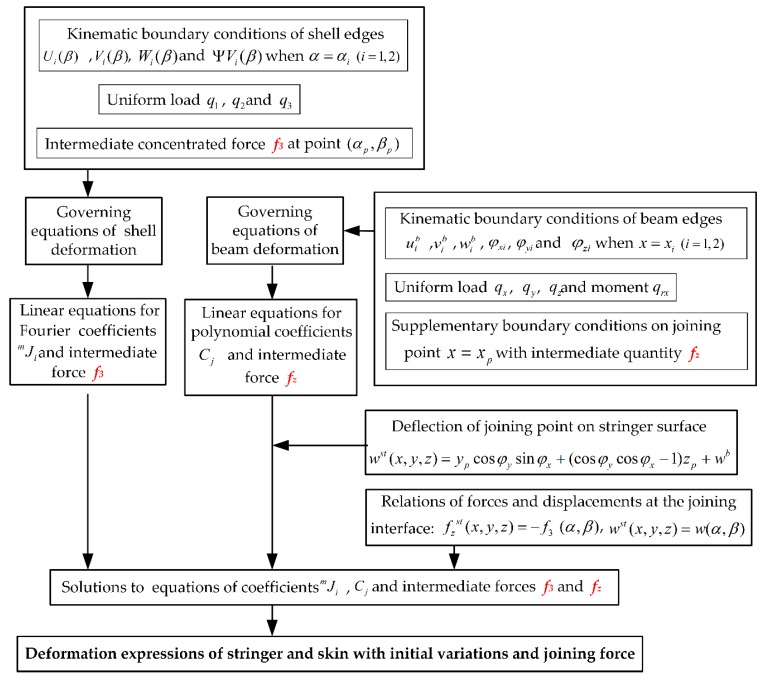
Procedure of panel components deformations with initial variation and joining force.

For solving the coefficients in the explicit expressions of stiffener and shell deformation, the proposed intermediate quantities, concentrated forces at the joining interface of the coupled structure as *f_i_* in Equation (20) and *f_y_* and *f_z_* in Equation (34), still should be calculated. Moreover, force *f_z_* and *f_3_* in the normal direction of the surface have the main effect on the actual panel profile since the automated drilling and riveting system of aircraft panel can achieve the measurement of surface normal and adjustment of machining direction. In summary, the detailed procedures for deformation calculation of stiffened panel with initial variation and joining force are illustrated in Figure 2.

## 3. Numerical Validation and Experiments

In this section, deformation propagation of fuselage stiffened panel with initial variations and mechanical interactions between the joined components are investigated. The semi-analytical calculation, FE simulation and practical experiments are performed by using the actual measured boundary conditions in engineering applications. Geometric and material properties of the stringer and shell are presented in Table 1 and Table 2.

### 3.1. Initial Kinematic Boundary Conditions of Panel Components

**Figure 3:**
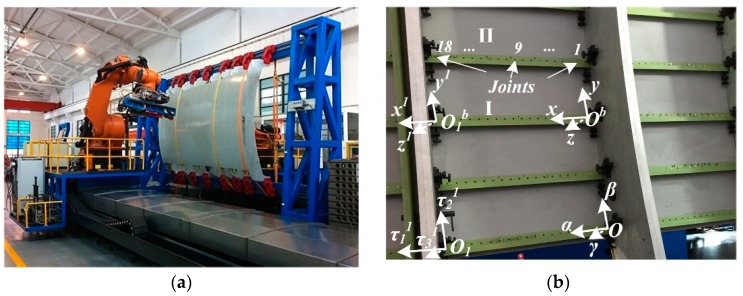
Experiment platform of aircraft fuselage panel assembly. (**a**) Aircraft assembly platform; (**b**) substructure of fuselage panel.

To determine the value of boundary conditions prescribed on the edges of stringers and skin, initial variations of points on the surfaces of fixturing boards, stringers I and II, and clamping mechanisms are captured by the measurement device in fuselage panel assembly platform, as shown in Figure 3.

The variations between the actual and theoretical position and orientation of the skin and stringers edges are the specified kinematic boundary conditions, as depicted in Table 3 and Table 4.

The measured variations are converted to the boundary conditions on curve edges of panel α=0 and α=495.5 with respect to curvilinear coordinate as the following expressions:

(36) $$u{|}_{\alpha =0}=v{|}_{\alpha =0}=w{|}_{\alpha =0}=\varphi_{v}{|}_{\alpha =0}=0,\;u{|}_{\alpha =495.5}=\Delta \tau_{1}-\alpha =-0.546,\;v{|}_{\alpha =495.5}=\Delta \tau_{2}\cdot \cos(\beta /R)-\Delta \tau_{3}\cdot \sin(\beta /R),\;w{|}_{\alpha =495.5}=\Delta \tau_{2}\cdot \sin(\beta /R)+\Delta \tau_{3}\cdot \cos(\beta /R),\;\;\varphi_{v}{|}_{\alpha =495.5}=\mathrm{atan}2(-{\Psi^{\prime }}_{31},\sqrt{{\left({\Psi^{\prime }}_{32}\right)}^{2}+{\left({\Psi^{\prime }}_{33}\right)}^{2}}),$$

where

(37) $${\Psi^{\prime }}_{}=\Psi (\beta /R,0,0)\Psi (\varphi_{1},\varphi_{2},\varphi_{3})\Psi (-\beta /R,0,0).$$

In addition to being imposed with the kinematic boundary constraints, the skin and stringers are also subjected to the gravity load. Load components of beam *q_x_*, *q_y_* and *q_z_* in the direction of local coordinate axes are 0, $-\rho^{b}gA\cos\left(b/2R-\theta_{b}\right)$ and $\rho^{b}gA\sin\left(b/2R-\theta_{b}\right)$, and $q_{rx}=0$. Load components of shell *q_1_*, *q_2_* and *q_3_* in the direction of local coordinate axes are 0, −ρghcos(β/R−b/2R) and −ρghsin(β/R−b/2R), respectively.

### 3.2. Initial Deformation of Panel Components

**Figure 4:**
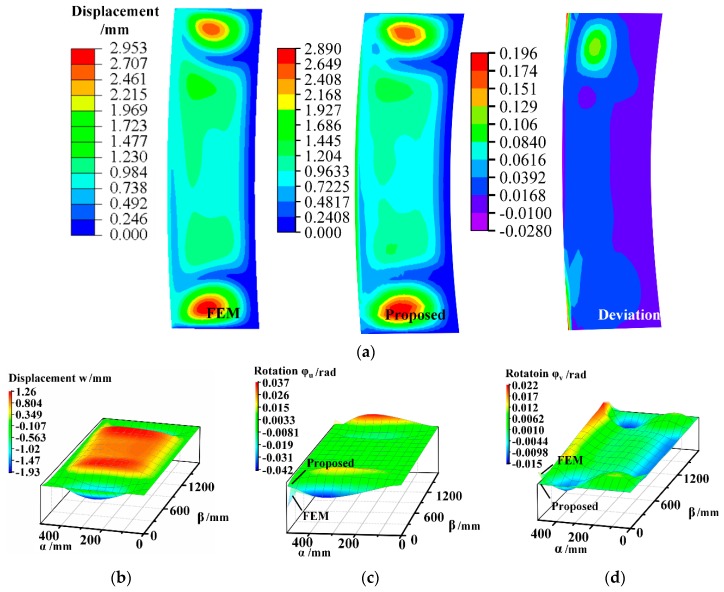
Displacement and rotation of the deformed shell middle surface. (**a**) Magnitude of displacement; (**b**) displacement in direction of γ; (**c**) rotation around axis α; (**d**) rotation around axis β.

**Figure 5:**
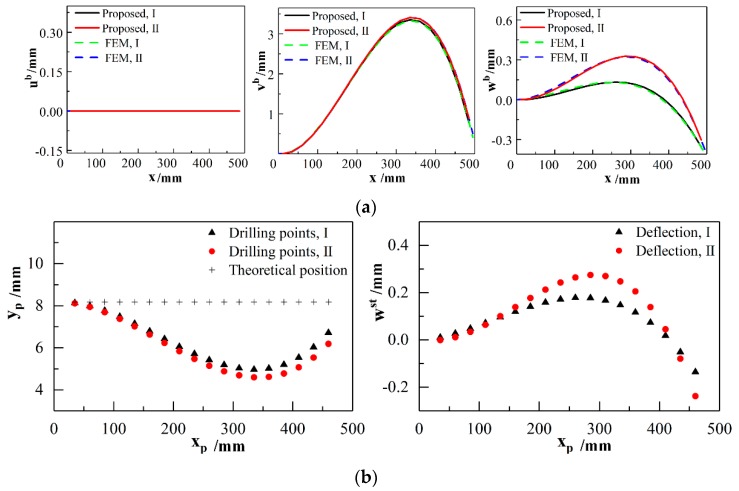
Deformation of stringers and presented deflections of points to be joined. (**a**) Deformation of beam I and II along the centroid locus of cross-section; (**b**) positions and deflection of drilling points on the stringers I and II.

During the positioning and clamping process, the panel components, skin and stringers, are just subjected to the initial kinematic boundary conditions listed in Equations (9) and (36) without the joining force. Substituting boundary conditions in Table 3 and Table 4 into the Fourier series expansion of shell displacement functions and beam deformation expressions, unknown coefficients Jmi and $C_{i}$ can be derived. Deformations of shell, beam I and beam II are provided by the proposed method, as shown in Figure 4 and Figure 5a. FE models of shell and beams are established by using commercial software ABAQUS^®^ 6.14 Standard with S8R element and B33 elements, respectively. Approximate element size is 25 mm and the grid number of FE model is over 1440.

**Figure 6:**
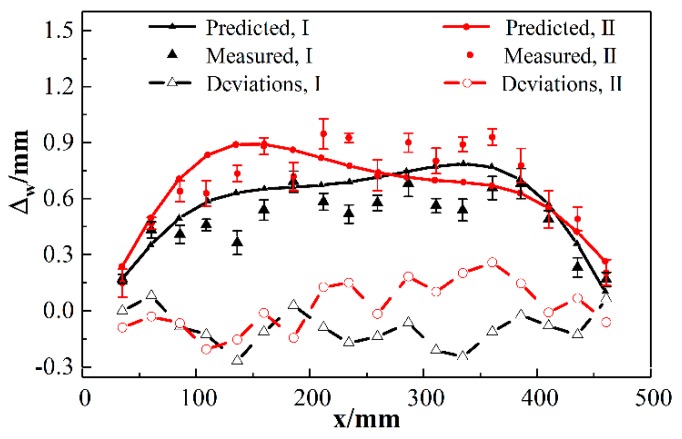
Clearances between the points to be joined on stringers and adjacent skin surface.

Besides, positions of points to be joined on the stringers I and II are predicted by solving a system of equations $u^{st}+x_{p}=x_{p}^{theoretical}$ and $v^{st}+y_{p}=y_{p}^{theoretical}$ by the Newton–Raphson approach. Substituting the solutions $\left(x_{p},y_{p}\right)$ and $z_{p}=13.062$ into Equation (35), deflections *w_st_* of the points to be joined are obtained, as shown in Figure 5b. Based on the theoretical arc lengths of skin, $\beta^{theoretical}=\theta_{b}R+l_{j}$, the actual positions of points to be joined on shell are obtained by solving a system of equations $u-\varphi_{v}h/2+\alpha_{p}=\alpha_{p}^{theoretical}$ and $v+\varphi_{u}h/2+\beta_{p}=\beta_{p}^{theoretical}$. Solutions $\left(\alpha_{p},\beta_{p}\right)$ are substituted into Equation (A2), deflections *w* of the points to be joined on skin are calculated. In the practical aircraft assembly process, actual clearance between the panel components $\Delta_{w}=w-w^{st}$ is measured to describe the relative deformation variation directly. A comparison of the predicted clearance and measured data is made to analyze the relative deformation components in the directions of surface normal, as shown in Figure 6.

Figure 4 and Figure 5 indicate that shell and beam deformation results provided by the proposed method are consistent with FEM result. As seen in Figure 4a, the shell displacement has increased nearly fourfold from initial variation 0.814 mm to 2.89 mm. Initial variations are obtained by $\sqrt{{\left(\Delta \tau_{1}-\alpha \right)}^{2}+\Delta \tau_{2}{}^{2}+\Delta \tau_{3}{}^{2}}$, the variables of which are listed in Table 3. The functional boundary conditions cause the dramatical changes in the displacement. Maximum deviation of shell displacement between FEM and the proposed results are 0.196 mm which can verify the accuracy of the semi-analytical solutions. Figure 4b shows that deflection *w* in the direction of surface normal increases considerably before a decline when β<0.2b and then decreases finally. Displacements *w* and rotation *φ_u_* distribute almost symmetrically with respect to section β=b/2. It is noted that *φ_u_* and *φ_v_* provided by the FEM have distortion near the edge node while the proposed semi-analytical solution has a more stable value, as seen in Figure 4c,d.

As seen in Figure 5a, displacements *w^b^* in *z*-axis direction have relatively smaller values compared with the large deflection in *y*-axis direction. When the drilling operation is conducted by the automated robot without compensation, the positions of drilling points are skewed, as shown in Figure 5b. The skewed drilling points would be joined in the subsequent assembly process, which seriously affects the product quality. Thus, the deformation prediction of stiffened aircraft panel components has a practical significance in aeronautical engineering.

It can be observed from Figure 4b, Figure 5b and Figure 6 that clearances *Δ*_w_ of the regions between stringers and skin have the similar trend with the displacements *w* of deformed shell on sections $\beta =\beta_{p}$. The major portion of clearance is the contribution from the skin deformation in the direction of surface normal. Absolute maximum deviations between predicted and measured clearances related to region I and II are 0.247 mm and 0.260 mm. Mean absolute deviations are 0.1127 mm and 0.1125 mm, respectively. It can be confirmed from the previous results that clearances provided by the proposed method and experimental approach have a good agreement.

### 3.3. Deformation of Stiffened Panel with Joints and Mixed Boundary Conditions

**Figure 7:**
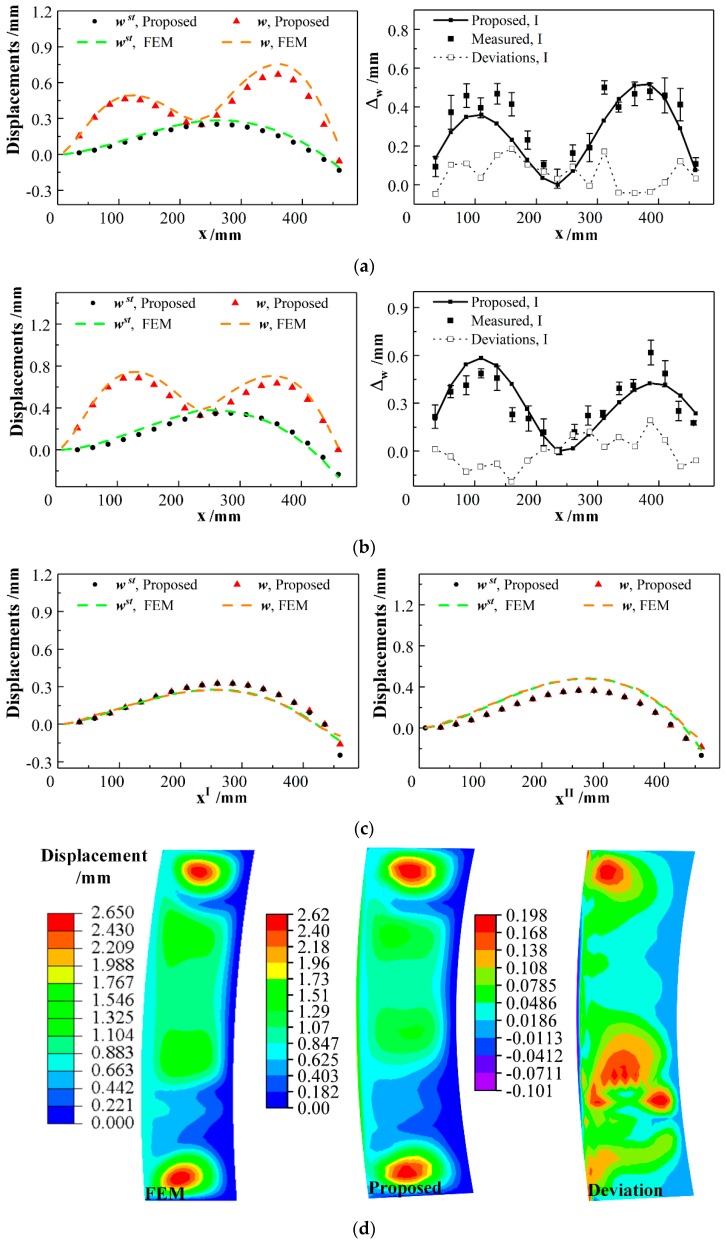
Deformation of stiffened panel with joints at different positions. (**a**) One joining point P_9_^I^ on stringer I; (**b**) one joining point P_9_^II^ on stringer II; (**c**) nine joining points on stringer I and nine joining points on stringer II; (**d**) magnitude of displacement given by FEM and proposed method and the deviation between both

Due to the mechanical interaction of the joined parts, the stringer–skin panel will suffer the secondary deformation in the joining process. Semi-analytical calculations and FE simulation of panel substructure model with different joining points are performed to analyze the deformation propagation of stiffened panel. Measurement experiments are conducted to verify the accuracy of the proposed method, as shown in Figure 3b. The intermediate quantity, concentrated force $f_{z}{}^{st}$ is calculated by semi-analytical solutions, as listed in Table 5. The displacements of joining points on the stringers and skin are predicted by solving unknown coefficients Jmi and $C_{i}$, as shown in Figure 7.

From Figure 7, it can be seen the joining operation causes dramatical deformation of shell and relatively slighter deformation of stringers. Figure 7a,b show that the absolute maximum deviations between the predicted and actual clearances are 0.180 mm and 0.193 mm when the point P_9_^I^ and P_9_^II^ are joined respectively. Displacements in the direction of surface normal provided by the proposed method and FEM are consistent with each other. Figure 7c,d indicates that the final positions of the stiffened panel with joints are similar to the stringers, which have higher stiffness than skin. Maximum deviation of shell displacement between FEM and the proposed results are 0.198 mm which can verify the accuracy of the semi-analytical solutions. Compared with the skin deformation shown in Figure 4a, the area of panel between two stiffeners deforms obviously with the joining operation conducted. Moreover, Table 5 shows the values of concentrated forces imposed on the same joining points, P_9_^I^ and P_9_^II^, change in different situations. Figure 7 illustrates that the clearances Δw have similar values when the points on the stringer I and II are joined simultaneously or separately, but the joining forces change a lot.

It can be concluded that the concentrated force on the joining point differs by the change of joining sequences. The relationship between the changing force and joining point position cannot be expressed by the discrete stiffness model which considers stiffeners just as immovable boundaries. Besides, discrete integration models need more zero and first spatial derivatives at the ends of individual segments to be re-entered into the deformation calculations when the joining force and clearances change. Moreover, compared with the curve interpolating algorithm, the proposed semi-analytical substructure model can provide the values of changing force with no more supplementary parameters introduced when the positions of joining points are different.

## 4. Conclusions

An efficient semi-analytical displacement solution of stiffened panel substructure model is proposed to analyze the deformation propagation of built-up aircraft panel in assembly processes. The proposed method takes into account the initial positioning variations, clamped boundary conditions imposed on stringers and skin, and the coupling deformation at the joining interface of panel components. The displacement and rotation expressions of the deformed stringers and skin are derived based on the modified Euler–Bernoulli beam theory and Kirchhoff–Love shell theory. The accuracy of the predicted deformation is confirmed by comparison with the FEM results and the measured data in the practical experiment of the aircraft panel assembly. The deviations between the experimental and proposed values of clearances between the deformed panel components are less than 0.260 mm in the positioning and clamping process. Moreover, the absolute maximum deviation between the predicted and actual clearances is 0.193 mm when the joining operations are conducted on the panel. The proposed model can provide the joining force values with no need of introducing more supplementary parameters when the positions of joining points are different. Thus, the proposed method is more effective to analyze the multi-component structure deformation.

## Figures and Tables

**Table 1:** Geometric and material property parameters of stringers.

Stringer	*a_s_*/mm	*l_j_*/mm	A/mm^2^	I_yy_/mm^4^	I_zz_/mm^4^	I_yz_/mm^4^	*E^b^*/GPa	*ρ^b^*/kg·m^−3^	*θ_b_*/rad
I	495	10	166.5	23420	14270	−14030	72	2830	0.13614
II									0.19897

**Table 2:** Geometric and material property parameters of skin.

*R*/mm	*a*/mm	*b*/mm	*h*/mm	*μ*	*E*/GPa	*ρ*/kg·m^−3^	*θ_g_*/rad
2724	495.5	1794.26	2	0.33	73	2780	1.2415

**Table 3:** Actual boundary conditions prescribed on shell curve edges with respect to Cartesian coordinate *Oτ_1_τ_2_τ_3_*.

*α*/mm	Δτ*_1_*/mm	Δτ*_2_*/mm	Δτ*_3_*/mm	*φ_1_*/rad	*φ_2_*/rad	*φ_3_*/rad
495.5	494.954	0.392835	−0.457491	−0.004490	−0.000188	−0.039646

**Table 4:** Actual boundary conditions prescribed on beam with respect to Cartesian coordinate *O^b^xyz*.

Stringer	*x*/mm	Δ*x*/mm	Δ*y*/mm	Δ*z*/mm	*φ_x_*/rad	*φ_y_*/rad	*φ_z_*/rad
I	10	0	0	0	0	0	0
	484.976	−3.8 × 10^−6^	0.781103	−0.356041	0.01601	0.005193	−0.039305
II	10	0	0	0	0	0	0
	485.013	3.1 × 10^−5^	0.891975	−0.302205	−0.01852	0.00765	−0.038902

**Table 5:** Calculated intermediate joining forces.

Point	Concentrated Forces/N
P_9_^I^	95.1882
P_9_^II^	104.734
$P^{I}{}_{2n+1}$, $P^{\mathrm{II}}{}_{2n+1}$(n=1⋯8)	103.89, 73.42, 37.73, 20.8, 15.24, 21.8, 33.15, 79.55, 61.45, 113.55, 92.09, 55.49, 30.64, 17.9, 14.57, 32.85, 28.99, 152.88

## Nomenclature

*a, b, R, h*	length of straight edge and arc edge, curvature radius, shell thickness, mm
*q_1_, q_2_, q_3_*	external load components in the directions of axes *τ_1_*, *τ_2_* and *τ_3_*, N·mm^−2^
*q_x_, q_y_, q_z_*	external load components along the axes *x*, *y* and *z*, N·mm^−1^
*q_rx_, q_ry_, q_rz_*	moment components about the axes *x*, *y* and *z*, N·mm
*f_1_, f_2_, f_3_*	concentrated force components caused by the joining interaction, N
*N_1_, N_2_, N_12_, N_21_, N_x_*	normal internal forces of shell and beam, N·mm^−2^
*M_1_, M_2_, M_12_, M_21_, M_y_, M_z_, M_yz_*	bending and twisting moments, N·mm
*u, v, w*	displacement components of local coordinate system *Oτ_1_τ_2_τ_3_*, mm
*φ_u_, φ_v_*	rotations around curvilinear coordinates, rad
*u^b^, v^b^, w^b^*	displacements along the centroid locus of the cross-section of beam, mm
*u^st^, v^st^, w^st^*	displacements of point P on the cross-section of the stringer, mm
*x_p_, y_p_, z_p_*	Coordinates of P with respect to Cartesian coordinate system *O^b^xyz*, mm
*σ_1_, σ_2_, σ_12_*	normal stresses and shear stress components, N·mm^−2^
E, E^b^	modulus of elasticity of shell and beam, GPa
μ, μ^b^	Poisson’s ratio
ρ, ρ^b^	density, kg·m^−3^
A, a_s_	cross-sectional area, mm^2^, length of the stiffener, mm
I_xx_, I_yy_, I_zz_, I_yz_, I_zy_	geometric torsional stiffness and moments of inertia for cross-section area, mm^4^

## Appendix A

All real and imaginary parts in complex roots of Equation (15) are given by

(A1) $$\xi_{1}=-k-k^{2}\sqrt{\frac{2b^{2}}{\sqrt{4b^{4}k^{4}+m^{4}\pi^{4}}-m^{2}\pi^{2}}},\;\xi_{2}=k+\sqrt{\frac{-m^{2}\pi^{2}+\sqrt{4b^{4}k^{4}+m^{4}\pi^{4}}}{2b^{2}}},\;\;\xi_{3}=-k+k^{2}\sqrt{\frac{2b^{2}}{\sqrt{4b^{4}k^{4}+m^{4}\pi^{4}}-m^{2}\pi^{2}}},\;\xi_{4}=k-\sqrt{\frac{-m^{2}\pi^{2}+\sqrt{4b^{4}k^{4}+m^{4}\pi^{4}}}{2b^{2}}}\;\mathrm{and}\;\;k=\sqrt[{4}]{3(1-\mu^{2})/\left(16h^{2}R^{2}\right)}.$$

Extended expressions for displacement functions are given as follows:

(A2) u=∑m=1∞sinm¯β{[h2(1−μ)24d6dα6−h2(2−μ)12m¯2d4dα4+h2(5−μ)24m¯4d2dα2−h212m¯6+1−μ2R2d2dα2]Φp1*+[μ−12R2(−m¯)ddα+(1+μ)h224m¯d5dα5−(1+μ)h212m¯3d3dα3+(1+μ)h224m¯5ddα]Φp2*−[1−μ2Rm¯2ddα+μ(1−μ)2Rd3dα3](Φp3*+Φhi*)}|α=αi,v=∑m=1∞cosm¯β{[(μ−1)m¯2R2d2dα−(1+μ)h2m¯24d5dα5+(1+μ)h2m¯312d3dα3−(1+μ)h2m¯524ddα]Φp1*+[1−μ2R2d2dα2+h212d6dα6−h2(5−μ)m¯224d4dα4+h2(2−μ)m¯412d2dα2−(1−μ)m¯22R2−h2(1−μ)m¯624]Φp2*+[(μ−1)(μ+2)m¯2Rd2dα2−(μ−1)m¯32R](Φp3*+Φhi*)}|α=αi,w=∑m=1∞sinm¯β{1−μ2R[−m¯2ddα−μd3dα3]Φp1*−[(μ−1)(μ+2)m¯2Rd2dα2+(1−μ)m¯32R]Φp2*+(1−μ2d4dα4+(1−μ)m¯42−(1−μ)m¯2d2dα2)(Φp3*+Φhi*)}|α=αi,φv=∑m=1∞sinm¯β{1−μ2R[m¯2d2dα2+μd4dα4]Φp1*+[(μ−1)(μ+2)m¯2Rd3dα3+1−μ2Rm¯3ddα]Φp2*+(−1−μ2d5dα5−1−μ2m¯4ddα+(1−μ)m¯2d3dα3)(Φp3*+Φhi*)}|α=αi,(i=1,2,3).

The Fourier series expansions of boundary conditions prescribed on shell edges are given by

(A3) Ui(β)=∑m=0∞Umsinm¯β, Vi(β)=∑m=0∞Vmcosm¯β, Wi(β)=∑m=0∞Wmsinm¯β and ΨVi(β)=∑m=0∞ΨmVsinm¯β,

where

(A4) Um={2b∫0bUi(β)sinm¯βdβm≠00m=0, Vm={2b∫0bVi(β)cosm¯βdβm≠012⋅2b∫0bVi(β)dβm=0, Wm={2b∫0bWi(β)sinm¯βdβm≠00m=0 and ΨmV={2b∫0bΨVi(β)sinm¯βdβm≠00m=0.

## References

[1] Tamijani A.Y., Kapania R.K. (2010). Buckling and Static Analysis of Curvilinearly Stiffened Plates Using Mesh-Free Method. AIAA J..

[2] Rodcheuy N., Frostig Y., Kardomateas G.A. (2017). Extended High-Order Theory for Curved Sandwich Panels and Comparison with Elasticity. J. Appl. Mech..

[3] Reddy J.N. (2010). Nonlocal Nonlinear Formulations for Bending of Classical and Shear Deformation Theories of Beams and Plates. Int. J. Eng. Sci..

[4] Fernandes R.R., Tamijani A.Y. (2017). Flutter Analysis of Laminated Curvilinear-Stiffened Plates. AIAA J..

[5] Su W., Cesnik C.E.S. (2011). Strain-Based Geometrically Nonlinear Beam Formulation for Modeling Very Flexible Aircraft. Int. J. Solids Struct..

[6] Carrera E., Pagani A., Petrolo M. (2013). Component-Wise Method Applied to Vibration of Wing Structures. J. Appl. Mech..

[7] Salehi M., Sideris P. (2018). A Finite-Strain Gradient-Inelastic Beam Theory and a Corresponding Force-Based Frame Element Formulation. Int. J. Numer. Methods Eng..

[8] Tamijani A.Y., Kapania R.K. (2012). Chebyshev-Ritz Approach to Buckling and Vibration of Curvilinearly Stiffened Plate. AIAA J..

[9] Carrera E., Zappino E. (2016). Carrera Unified Formulation for Free-Vibration Analysis of Aircraft Structures. AIAA J..

[10] Sobota P.M., Dornisch W., Muller R., Klinkel S. (2017). Implicit Dynamic Analysis Using an Isogeometric Reissner-Mindlin Shell Formulation. Int. J. Numer. Methods Eng..

[11] Ventsel E., Krauthammer T. (2001). Thin Plates and Shells: Theory, Analysis, and Applications.

[12] Zappino E., Carrera E. (2018). Multidimensional Model for the Stress Analysis of Reinforced Shell Structures. AIAA J..

[13] Guida M., Marulo F., Abrate S. (2018). Advances in Crash Dynamics for Aircraft Safety. Prog. Aerosp. Sci..

[14] Silva P.B., Mencik J.M., Arruda J.R.D. (2016). Wave Finite Element-Based Superelements for Forced Response Analysis of Coupled Systems via Dynamic Substructuring. Int. J. Numer. Methods Eng..

[15] Pacheco D.R.Q., Marques F.D., Ferreira A.J.M. (2018). Finite Element Analysis of Fluttering Plates Reinforced by Flexible Beams: An Energy-Based Approach. J. Sound Vib..

[16] Slemp W.C.H., Kapania R.K., Mulani S.B. (2010). Integrated Local Petrov-Galerkin Sinc Method for Structural Mechanics Problems. AIAA J..

[17] Sapountzakis E.J., Mokos V.G. (2008). An Improved Model for the Analysis of Plates Stiffened by Parallel Beams with Deformable Connection. Comput. Struct..

[18] Ahmad N., Kapania R.K. (2016). Free Vibration Analysis of Integrally Stiffened Plates with Plate-Strip Stiffeners. AIAA J..

[19] Timoshenko S., Woinowsky-Krieger S. (1959). Theory of Plates and Shells.

[20] Wang Q., Hou R., Li J., Ke Y. (2018). Analytical and Experimental Study on Deformation of Thin-Walled Panel with Non-ideal Boundary Conditions. Int. J. Mech. Sci..

[21] Carrera E., Giunta G., Petrolo M. (2011). Beam Structures: Classical and Advanced Theories.

[22] Przemieniecki J.S. (1968). Theory of Matrix Structural Analysis.

[23] Shabana A.A., Yakoub R.Y. (2001). Three Dimensional Absolute Nodal Coordinate Formulation for Beam Elements: Theory. J. Mech. Des..

[24] Wang Q., Hou R., Li J., Ke Y., Maropoulos P.G., Zhang X. (2018). Positioning Variation Modeling for Aircraft Panels Assembly Based on Elastic Deformation Theory. Proc. Inst. Mech. Eng. Part B J. Eng. Manuf..

